# Psychological Determinants of Purchasing Behavior Among Individuals Indifferent to Reduced-Salt Products

**DOI:** 10.3390/nu18111800

**Published:** 2026-06-03

**Authors:** Yasunori Akamatsu, Misako Nakadate, Nanae Tanemura, Masuko Kobori

**Affiliations:** 1 National Institute of Health and Nutrition, National Institutes of Biomedical Innovation, Health and Nutrition, 3–17 Senriokashimmachi, Settsu 566-0002, Osaka, Japan; 2 Food and Health Science Research Institute, Nissui Corporation, 1-3-1 Nishishimbashi, Minato-ku, Tokyo 105-8676, Japan

**Keywords:** behavior change, salt intake, reduced-salt products, integrated behavioral model, individuals indifferent to reduced-salt products

## Abstract

**Background/Objectives:** To promote salt reduction before health problems arise, it is important to understand factors associated with reduced-salt product purchasing among consumers with low interest in such products. This study examined psychological determinants of intention to purchase reduced-salt products among Japanese adults who were not actively purchasing them. **Methods:** An exploratory sequential mixed-methods design was used, including qualitative interviews followed by a cross-sectional web-based survey of 800 men and women aged 18–59 years in Japan. Participants were categorized into precontemplation and contemplation stages based on the transtheoretical model. Associations between purchase intention and three Integrated Behavioral Model categories—attitude, perceived norm, and personal agency—were examined. Percentage-to-gain values were calculated for each belief item. **Results:** All three category scores were significantly associated with purchase intention, with attitude showing the strongest association (OR = 12.56, 95% CI: 6.93–22.79). In stratified analysis, attitude showed a stronger association in the precontemplation stage (OR = 18.40, 95% CI: 8.51–39.78), whereas no category score was significantly associated with purchase intention in the contemplation stage. In the precontemplation stage, relatively high percentage-to-gain values were observed for holistic wellness-oriented beliefs and product availability in usual supermarkets. **Conclusions:** Attitude was most strongly associated with intention to purchase reduced-salt products, particularly in the precontemplation stage. Holistic wellness-oriented beliefs and product availability may represent promising targets for future communication or food environment interventions. These findings may provide a basis for future studies testing strategies to increase actual purchases of reduced-salt products and reduce salt intake.

## 1. Introduction

Excessive salt intake is closely associated with hypertension, a major risk factor for noncommunicable diseases [1,2]. The World Health Organization (WHO) recommends a daily salt intake of less than 5 g. However, the global average intake remains significantly higher at 10.8 g per day [3]. In Japan, the average intake for men aged 20 and over was 10.7 g per day in 2023, and 9.1 g per day for women [4], approximately double the WHO target. These intake levels indicate that high salt intake remains a significant public health challenge in Japan because of its association with hypertension and other noncommunicable diseases, particularly in the context of a rapidly aging population. Some unprocessed foods also naturally contain salt. Examples include milk, eggs, meat, fish, shellfish, and some vegetables [5]. However, salt reduction strategies often focus on modifiable sources of added salt, including seasonings and processed or prepared foods.

Salt reduction education initiatives are being implemented globally and include nutrition counseling and individual consultations aimed at reducing salt intake. Educational interventions for behavioral change have been reported to be effective in reducing salt intake [6]. In Japan, studies conducted since 2000 on educational interventions for salt reduction have also reported a decrease in salt intake among hypertensive patients [7,8,9] and healthy middle-aged and older adults [10]. A study that targeted healthy middle-aged and older adults [10] noted that participants voluntarily responded to a public recruitment call for the study. This age group already had a high level of awareness regarding salt reduction, which likely contributed to the success of the behavioral change. Therefore, salt reduction education is considered an effective behavioral change intervention for individuals interested in their health, particularly those affected by aging or hypertension. However, knowledge-based education may be less effective for individuals without hypertension or immediate health concerns because they may not perceive salt reduction as personally relevant. Therefore, different approaches may be needed for consumers with low interest in salt reduction.

A large-scale international survey of approximately 7000 members of the general population aged 18–65 without serious illnesses reported that one-third of the participants showed no interest in salt reduction and tended to underestimate their salt intake [11]. In Japan, the market size for health-related products, including reduced-salt products, has continued to expand in recent years, indicating rising public health awareness. However, Japanese people show less interest in salt reduction compared to people in other countries, with awareness particularly low in younger age groups [12]. Furthermore, a recent study reported that the preferred appeal expressions on reduced-salt product packaging differ between those interested in salt reduction and those who are not, suggesting that different approaches are needed based on varying levels of interest [13].

Therefore, to promote salt reduction before health problems arise, it is important to understand factors associated with reduced-salt product purchasing among consumers who are not actively purchasing such products. In particular, clarifying the psychological and cognitive tendencies toward reduced-salt products among such consumers may help inform future communication and food environment strategies.

Purchasing reduced-salt products is influenced by a complex interplay of individual taste preferences, personal health conditions, and food environments, and can be regarded as a dietary behavior that may be affected by psychological, cultural, and social factors. The theory of planned behavior [14] and the capability, opportunity, motivation–behavior model [15] are also relevant frameworks for understanding health-related behavior. By comparison, the integrated behavioral model (IBM) [16,17,18] is considered particularly useful for explaining health-related behaviors that may be influenced by psychological, social, and cultural contexts. IBM has been used to explain behaviors such as willingness to receive influenza [19] and COVID-19 [20] vaccinations or taking HIV prevention measures [21] and to design policies and public health campaigns [22]. Furthermore, studies have also used IBM to predict vegetable and fruit consumption, demonstrating its usefulness in predicting and explaining dietary behavior [23]. The IBM consists of three constructs that influence behavioral intention: attitude, perceived norm, and personal agency. Attitude refers to an individual’s overall evaluation of a behavior, whether favorable or unfavorable. Perceived norm refers to the influence an individual perceives from society or others regarding a behavior. Personal agency refers to an individual’s perceived ability to perform a behavior. By classifying beliefs about a behavior into these categories and examining their strength in relation to behavioral intention, IBM can be used to account for psychological factors that shape intention toward the behavior. Therefore, IBM was considered an appropriate framework for examining reduced-salt product purchasing behavior in this study.

Furthermore, identifying specific beliefs that may represent promising targets is important for developing future communication or food environment interventions related to reduced-salt product purchasing. A percentage-to-gain analysis estimates the potential change in behavioral intention that could be expected if individuals who do not yet hold a belief came to hold it through external messaging [24]. It has been used in designing communications for social marketing campaigns aimed at preventing tobacco [25] and e-cigarette [26] use among youth and increasing trust in COVID-19 vaccines [27]. In this study, this method was used to identify specific beliefs within the three IBM categories that showed high potential as targets for future interventions related to purchase intention.

Therefore, this study aimed to examine psychological determinants of intention to purchase reduced-salt products among individuals who do not actively purchase such products. Accordingly, this study addressed the following research questions (RQs):

- RQ1: Which IBM category is most strongly associated with purchase intention for reduced-salt products?
- RQ2: Which specific beliefs show the highest percentage-to-gain values as potential targets for future communication or food environment interventions?

By focusing on individuals with low interest in reduced-salt products, this study may contribute to the development of strategies for engaging consumers with low health concerns or low motivation toward salt reduction.

## 2. Materials and Methods

**Figure 1:**
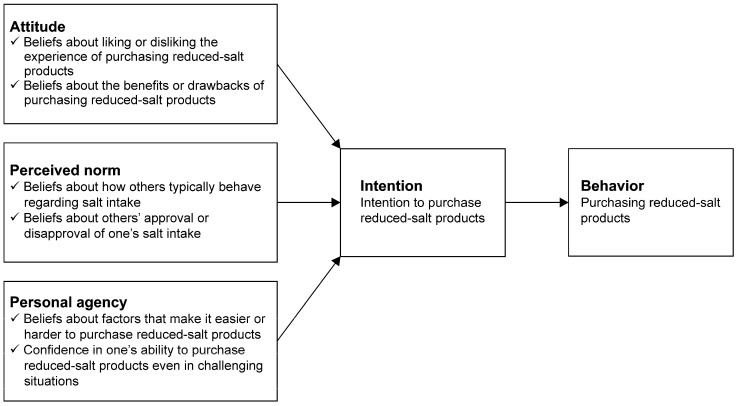
Schematic presentation of the integrated behavioral model in this study.

**Figure 2:**
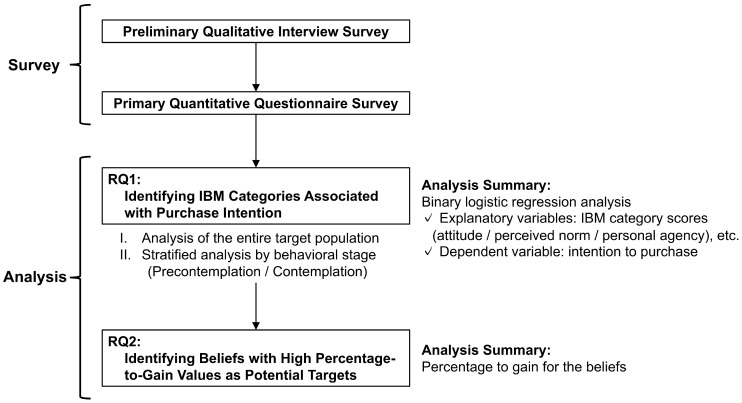
Study flow.

This study used an exploratory sequential mixed-methods design. Preliminary qualitative interviews were conducted to extract beliefs corresponding to the IBM categories, followed by a quantitative questionnaire survey and percentage-to-gain analysis. Figure 1 illustrates behavioral intention and the three IBM categories (attitude, perceived norm, and personal agency) as applied in this study. The flow of this study is illustrated in Figure 2.

The transtheoretical model is useful for classifying individuals according to their readiness for behavior change [28]. Based on this model, this study defined the behavioral stages related to purchasing reduced-salt products among individuals not actively purchasing such products as follows:

Purchasing reduced-salt products within the next six months:

- Precontemplation stage: not actively planning to purchase.
- Contemplation stage: actively planning to purchase.

### 2.1. Preliminary Qualitative Interview Survey

#### 2.1.1. Study Participant and Selection Process

The survey was conducted under contract with a marketing research company (Neo Marketing, Inc., Tokyo, Japan). First, a screening questionnaire was administered to the research company’s panel to select the interview participants. Subsequently, online interviews were conducted with the participants. The selection and exclusion criteria for interview participants were set as follows:

Selection criteria:

1. Healthy men and women aged 18 to 59 residing in Japan;
2. Individuals not actively purchasing reduced-salt products.

Exclusion criteria:

1. Individuals who self-identify as having slightly elevated blood pressure *;
2. Individuals who have been diagnosed with hypertension.

* Guideline for slightly elevated blood pressure: systolic blood pressure ≥ 130 mmHg or diastolic blood pressure ≥ 85 mmHg [29].

Interview participants were classified into eight segments based on their behavioral stage regarding reduced-salt product purchase (precontemplation stage/contemplation stage), presence/absence of family members with hypertension living in the same household, and age group (18–39 years old/40–59 years old). The standard sample size for interpretive phenomenological interviews is approximately six participants per segment [30]. Therefore, the target sample size per segment was six, resulting in a total of 48 interview participants. To ensure balanced gender ratios, the number of participants was adjusted to achieve a 1:1 ratio of men and women in each segment (three men and three women). Interviews were conducted between 15 January and 3 February 2024.

#### 2.1.2. Screening Questionnaire

The screening questionnaire collected interview participants’ background information, including selection, exclusion, and segmentation criteria. The background information included gender, age, occupation, household size, household annual income, blood pressure status, presence/absence of chronic diseases other than hypertension, current purchasing status of reduced-salt products, and behavioral stage regarding the purchase of reduced-salt products. The study did not collect personal information, only the interview participants’ responses.

#### 2.1.3. Interviews

Interviews were conducted using semi-structured interview guidelines that outlined the questions in advance and focused on themes corresponding to the three IBM categories. Each interview lasted approximately 30 min. All the interviews were transcribed. Using an interpretive phenomenological approach [31], we interpreted narratives and extracted beliefs, focusing on patterns, discrepancies, and inconsistencies. To eliminate researcher bias, two analysts independently reviewed the interpretations and checked the results for consistency. When differing interpretations arose, the two analysts discussed them and adjusted the content of the interpretations. To minimize omission of potentially important minority perspectives, even beliefs mentioned by only a single participant were retained without exclusion. Consequently, all 48 extracted beliefs were included as evaluation targets in the subsequent quantitative survey.

### 2.2. Primary Quantitative Questionnaire Survey

#### 2.2.1. Study Participant and Selection Process

The survey was conducted under a contract with a marketing research company (Cross Marketing, Inc., Tokyo, Japan). First, a screening questionnaire provided by the research company was administered to the panel to select respondents for the main survey questionnaire. Subsequently, the main survey questionnaire was administered to respondents who met the inclusion criteria. For screening, enrollment, and the main survey questionnaires, we used a series of web-based response systems developed by the research company. Responses to the screening questionnaire were immediately evaluated for eligibility according to the participant criteria. Only those who met the inclusion criteria participated in the main survey. The inclusion criteria for the main survey participants were as follows:

Selection criteria:

1. Men and women aged 18 to 59 residing in Japan;
2. Individuals not actively purchasing reduced-salt products.

Exclusion criteria:

1. Individuals who perceive themselves to have elevated blood pressure *;
2. Individuals who have been diagnosed with hypertension.

* Guideline for elevated blood pressure: systolic blood pressure ≥ 130 mmHg OR diastolic blood pressure ≥ 80 mmHg [32].

The target sample size was 384 individuals, assuming a 50% response rate, 5% margin of error, and a 95% confidence level. Thus, the target number of respondents for the survey was 400 men and 400 women, for a total of 800 individuals. Population distribution ratios based on the Ministry of Internal Affairs and Communications Statistics Bureau’s prefectural population estimates [33] were applied to weight the number of participants per region. The survey was concluded once the target number of participants per region was reached. The survey was conducted from 17 to 19 January 2025.

#### 2.2.2. Screening Questionnaire

The questionnaire primarily gathered information on the inclusion criteria for the main survey, including gender, age, region of residence, blood pressure status, current purchasing of reduced-salt products, and current behavioral stage regarding reduced-salt product purchasing (precontemplation stage/contemplation stage).

#### 2.2.3. Main Survey Questionnaire

The questionnaire collected background information and information regarding reduced-salt product purchasing behavior. Background information included occupation, household size, household annual income, and presence/absence of chronic diseases other than hypertension. Regarding information on reduced-salt product purchasing behavior, we asked about the presence/absence of intention to purchase reduced-salt products and requested ratings on a 5-point scale (1 = Strongly disagree; 2 = Disagree; 3 = Neither agree nor disagree; 4 = Agree; 5 = Strongly agree) for the level of agreement with 48 belief items extracted through the interviews. The contents of 48 belief items are shown in Table S1.

### 2.3. Analysis

#### 2.3.1. IBM Category Scores

A summary of the belief items is shown in Table 1. IBM category scores were calculated as follows. First, for the participants’ 5-point scale responses, belief items 1–9 and 21–48 were directly converted to scores of 1–5. For belief items 10–20, which represent negative beliefs, such as perceived disadvantages or lack of need for reduced-salt products, and thus have an opposite orientation to other belief items, the numerical values were inverted during scoring (e.g., a rating of 1 became a score of 5). Mean scores were calculated for each category (attitude, perceived norm, and personal agency) and became the IBM category scores for each participant.

Cronbach’s alpha was calculated for each IBM category to verify internal consistency. The Cronbach’s alpha values for attitude (20 items), perceived norm (12 items), and personal agency (16 items) were 0.62, 0.80, and 0.95, respectively. Considering the relatively low internal consistency of the attitude construct, an exploratory factor analysis (EFA) of the 20 attitude belief items was additionally conducted using the generalized least squares method with Varimax rotation. The factor loading pattern is presented in Table S2. The EFA indicated a four-factor structure; however, the factor loading pattern did not show a clear separation between the experiential aspect of attitude, such as liking or disliking the experience of purchasing reduced-salt products, and the instrumental aspect, such as perceived benefits or drawbacks, as illustrated in Figure 1. Therefore, the EFA results were interpreted as supplementary evidence of heterogeneity within the attitude belief items, rather than as a basis for constructing post hoc attitude subscales. Because the primary purpose of this study was to examine which IBM categories were associated with purchase intention, attitude was retained as a single IBM category in the main analyses, consistent with the theoretical structure of the IBM framework.

#### 2.3.2. Stratified Analysis by Behavioral Stage

An analysis was first conducted on the entire sample, followed by a stratified analysis by behavioral stage (precontemplation/contemplation). Table S3 shows the characteristics of the precontemplation and contemplation stages and their information on reduced-salt product purchasing behavior. Approximately 90% of the individuals in the contemplation stage expressed purchase intention and were ready to move toward purchase behavior. In contrast, about 80% of those in the precontemplation stage lacked purchase intention. Furthermore, the IBM category scores were significantly higher in the contemplation stage than in the precontemplation stage. The results suggested that IBM category scores might be influenced by differences in the distribution of the purchase intention. Therefore, we performed a stratified analysis by behavioral stage.

#### 2.3.3. Identifying IBM Categories Associated with Purchase Intention

We performed binary logistic regression analysis and calculated the odds ratio (OR) for the intention to purchase reduced-salt products based on the IBM category scores (attitude, perceived norm, and personal agency) to determine which IBM categories were significantly associated with purchase intention. This approach was selected because RQ1 aimed to compare the strength of association between each IBM category score and the presence of purchase intention, while treating purchase intention as a dichotomous outcome. The dependent variable was intention to purchase reduced-salt products (1 = present; 0 = absent), with 0 = absent as the reference category. The primary explanatory variable was the IBM category score. The OR indicates how many times greater the odds of purchase intention are when the IBM category score increases by 1.

The analysis models were set as follows:

- Model 1: Each IBM category score individually.
- Model 2: Each IBM category score adjusted for age and gender.

When constructing the analysis model, age was adopted as a control variable owing to the possible differences in purchasing orientation toward reduced-salt products by age group. Furthermore, gender was adopted because, as shown in Table 2, a significant difference in average IBM scores between men and women was observed. In addition to age and gender, we examined sociodemographic variables such as region of residence, occupation, household size, household annual income, and the presence of chronic diseases other than hypertension, as well as combinations of these variables. However, none of these variables showed a significant association with intention to purchase reduced-salt products. The final models were defined as Model 1, which included only the IBM category score, and Model 2, which included the IBM category score adjusted for age and gender.

#### 2.3.4. Sensitivity Analysis

For the sensitivity analysis, we excluded participants who reported chronic diseases other than hypertension and repeated the binary logistic regression analyses described in Section 2.3.3. The same Model 1 and Model 2 specifications were applied as in the primary analyses.

#### 2.3.5. Identifying Promising Beliefs Using Percentage-to-Gain Analysis

Following Hornik & Woolf’s percentage-to-gain analysis method, we calculated the percentage to gain for each belief item [24]. This percentage is an estimate that indicates the maximum expected effect, calculated as the difference between the proportion of the individuals holding one belief who also have behavioral intention and the proportion of the entire target group who have behavioral intention. For belief items 1–9 and 21–48, we calculated the difference between the proportion of those intending to purchase reduced-salt products within the group that responded “5 = Strongly agree” or “4 = Agree” and the proportion of those who intended to purchase reduced-salt products within the entire target population. For belief items 10–20, we calculated the difference between the proportion of respondents who intended to purchase reduced-salt products within the group that answered “1 = Strongly disagree” or “2 = Disagree” and the proportion of respondents who intended to purchase reduced-salt products within the entire sample.

#### 2.3.6. Other Statistical Analyses

We performed an unpaired *t*-test to compare the two groups’ means. To compare the distribution of numbers, we performed Fisher’s exact test for 2 × 2 cases and the chi-square test for other cases. All the statistical analyses were performed using IBM SPSS Statistics version 29 (IBM Corporation, Armonk, NY, USA). A two-tailed *p*-value less than 0.05 was considered statistically significant.

### 2.4. Ethics Approval and Informed Consent

For the preliminary qualitative and the primary quantitative surveys, participants were provided with an explanatory webpage detailing the study’s design and objectives prior to starting the screening questionnaire. This was done to ensure participants understood the purpose of the research. Informed consent was obtained beforehand. For interview participants, the study was explained again before the interview, and consent was obtained once more. Participation in the survey was considered voluntary based on ethical standards guidelines published in Japan. This study was conducted following a review by the Ethics Review Committee of the National Institutes of Biomedical Innovation, Health and Nutrition (Preliminary Qualitative Interview Survey: Approval No. 2023-022, Approval Date: 3 October 2023; Primary Quantitative Questionnaire Survey: Approval No. 2024-043, Approval Date: 29 November 2024). The survey was conducted in accordance with the ethical principles established in the Declaration of Helsinki.

## 3. Results

### 3.1. Preliminary Qualitative Interview Survey

Table S4 summarizes the characteristics of the 48 interview participants who were not actively purchasing reduced-salt products. We conducted interviews with the participants regarding the purchase of reduced-salt products and extracted beliefs corresponding to three IBM categories (attitude, perceived norm, and personal agency) from the interview participants’ statements. This yielded 48 belief items (Table S1). These belief items comprised 20 attitude items (9 positive, 11 negative), 12 perceived norm items, and 16 personal agency items.

### 3.2. Primary Quantitative Questionnaire Survey

Table 2 shows the characteristics (current behavioral stage and background information) and information regarding reduced-salt product purchasing behavior (presence of the purchase intention and IBM category scores) of 800 participants (400 men and 400 women) across Japan who were not actively purchasing reduced-salt products. Regarding the behavioral stage, 335 men and 314 women were in the precontemplation stage. Thus, for both genders, approximately 80% of those not currently purchasing reduced-salt products were uninterested individuals (precontemplation stage) not actively planning to purchase within 6 months, while approximately 20% were interested individuals (contemplation stage) actively planning to purchase within 6 months. Statistically significant differences between genders were observed in age, occupation, household size, household annual income, purchase intention, and IBM category scores.

### 3.3. RQ1: Which IBM Category Is Most Strongly Associated with Purchase Intention for Reduced-Salt Products?

Table 3 shows the results of the binary logistic regression analysis examining the association between the IBM category scores and the intention to purchase reduced-salt products. By examining whether attitude, perceived norm, and personal agency were individually associated with purchase intention for reduced-salt products, we evaluated which IBM category was most strongly associated with purchase intention among individuals who were not currently purchasing such products.

In Model 1, all three category scores were significantly related to intention to purchase reduced-salt products (attitude: OR = 13.08, *p* < 0.001; perceived norm: OR = 1.72, *p* < 0.001; personal agency: OR = 3.74, *p* < 0.001). Among the three category scores, attitude showed the largest OR. Similarly, in Model 2 adjusted for age and gender, all three scores were significantly related to intention (attitude: OR = 12.56, *p* < 0.001; perceived norm: OR = 1.67, *p* < 0.001; personal agency: OR = 3.65, *p* < 0.001), with the OR for attitude being the largest.

Next, we present the results of a series of stratified analyses by behavioral stage. IBM category scores were compared between groups with and without purchase intention in precontemplation and contemplation stages (Table 4). In the precontemplation stage, the scores for attitude, perceived norm, and personal agency were all significantly higher in the group with purchase intention than in the group without purchase intention. By contrast, no significant differences were observed between the two groups in the contemplation stage.

Binary logistic regression analyses were performed to assess the relationship between IBM category scores and the intention to purchase reduced-salt products at each behavioral stage. Table 5 summarizes the results. The analytical model is the same as the model listed in Table 3. In the precontemplation stage, both Models 1 and 2 showed significant associations between attitude, perceived norm, personal agency, and purchase intention. Notably, the attitude score had the largest OR, exceeding 18 in Models 1 and 2. Conversely, in the contemplation stage, no significant associations were found for any scores in either model. Therefore, the IBM categories were more clearly associated with purchase intention in the precontemplation stage than in the contemplation stage. In particular, attitude showed the strongest association with purchase intention in the precontemplation stage.

Sensitivity analyses were conducted after excluding participants who reported chronic diseases other than hypertension. The results are presented in Supplementary Table S5. These results were broadly consistent with those of the main analyses previously described. In both Model 1 and Model 2, the direction of the associations remained unchanged. The strong association between attitude and intention to purchase reduced-salt products was maintained in the overall sample and the precontemplation stage.

### 3.4. RQ2: Which Specific Beliefs Show the Highest Percentage-to-Gain Values as Potential Targets for Future Communication or Food Environment Interventions?

We calculated the percentage to gain for each belief item. The percentage is an estimate indicating the maximum expected effect, calculated as the difference between the proportion of individuals holding one belief who also possess behavioral intention and the proportion of the entire target group who possess behavioral intention. To summarize, it compares how the number of people with behavioral intention would change (increases if the value is positive, decreases if negative) if the entire target group were to come to hold that belief (or, for belief items 10–20, were not to come to hold it). We used the percentage to identify beliefs that may represent promising targets related to purchase intention.

For each belief item, the percentage to gain was calculated separately for the precontemplation and contemplation stages by gender (Table S6). In the contemplation stage, the highest percentages to gain were 9.0% for men and 8.1% for women, with no belief items exceeding 10%. In contrast, in the precontemplation stage, 13 belief items for men and 15 for women had percentages to gain of 10% or higher. Therefore, compared to the precontemplation stage, fewer beliefs influenced purchase intention in the contemplation stage, and their impact was smaller.

Table 6 lists the top five belief items by percentage to gain in the precontemplation stage. For both genders, Belief item 9 (attitude: “Using reduced-salt products gives me peace of mind that I am eating something that looks good for me”), Belief item 41 (personal agency: “I would buy reduced-salt products if they were available in the supermarkets I usually go to”), and Belief item 44 (personal agency: “I want to consciously use reduced-salt products when I am feeling disordered in my diet”) ranked. These results identified beliefs that may represent promising targets for individuals in the precontemplation stage.

## 4. Discussion

To the best of our knowledge, this study provides new evidence on psychological factors associated with intention to purchase reduced-salt products among individuals who are not actively purchasing such products. The results of the IBM analysis showed that attitude was most strongly associated with purchase intention, particularly among individuals in the precontemplation stage. The percentage-to-gain analysis identified several beliefs that may serve as promising targets for future communication or food environment interventions. These findings provide a new perspective for designing promotional campaigns and food environment strategies for consumers who are currently indifferent to reducing their salt intake.

When classifying the study participants (individuals not currently actively purchasing reduced-salt products) into two behavioral stages, approximately 80% of both men and women were in the precontemplation stage (not actively planning to purchase within six months) (Table 2). Our results indicate that the majority of individuals who do not actively purchase reduced-salt products are not interested in purchasing them.

In the literature on salt-reduction behavior, the proportion of individuals in the precontemplation stage varied significantly across studies and countries. Previous Japanese research reported approximately 77% [34], while findings in other countries ranged between 33% and 84% [11,35,36]. Purchasing reduced-salt products is not synonymous with reduced-salt behavior; consequently, simple comparisons should be made cautiously when viewed from a broader perspective of salt reduction. The current study’s finding that approximately 80% of individuals who were not purchasing reduced-salt products were uninterested in purchasing them is largely consistent with prior Japanese research [34]. Together, these findings indicate that the proportion of individuals with low interest in salt-reduction-related behavior remains high in Japan compared with findings from other countries, highlighting the need for approaches that can engage individuals with low interest in salt reduction or reduced-salt products.

Regarding RQ1, the results in Table 3 indicate that, among all participants, attitude, perceived norm, and personal agency were all associated with purchase intention, with attitude showing the strongest relationship. The sensitivity analyses excluding participants with chronic diseases other than hypertension showed a similar pattern (Table S5). Previous studies using the theory of reasoned action model with British women [37] and the theory of planned behavior model with Iranian pregnant women [38] have reported an association between reduced salt intake and attitudes. Although the participants, behaviors, and behavioral models differed, their significant associations with attitudes aligned with the current study’s findings. This consistency suggests that attitude is relevant across different salt-reduction-related contexts, while the present study may further extend this evidence by showing its strong association with intention to purchase reduced-salt products among individuals with low interest in such products.

In the stratified analysis, significant associations were observed between each IBM category score and purchase intention in the precontemplation stage, and the association with attitude was even stronger than in the entire sample. However, no such association was observed in the contemplation stage (Table 5). In this study, the contemplation stage also included individuals in the preparation stage (actively planning to purchase within the next month). Therefore, it is possible that some individuals were already transitioning to purchasing behavior, which influenced the IBM category scores and resulted in inconsistent associations with purchase intention.

Table S3 shows no differences in background factors, such as age, gender, or household income, between the precontemplation and contemplation stages. These findings suggest that, among individuals in the precontemplation stage, intention to purchase reduced-salt products was more strongly associated with the measured psychological factors than with the sociodemographic variables examined in this study.

In IBM, attitude is defined as an individual’s overall evaluation of a behavior, whether favorable or unfavorable, and is composed of affective/experiential and cognitive/instrumental aspects [16,17,18]. Food preferences are believed to be based on dietary experiences during childhood [39], and the same is thought to apply to saltiness [40]. As individuals age, their knowledge of the relationship between salt intake and health, such as its impact on hypertension, increases. These experiential preferences and health-related knowledge may shape attitudes toward purchasing reduced-salt products through both affective/experiential and cognitive/instrumental aspects. Differences in attitudes formed through these processes may influence individuals’ behavioral stage regarding the purchase of reduced-salt products.

A recent study on packaging expressions reported that among those interested in salt reduction, packaging that clearly states “XX% salt reduction” is perceived more favorably [13]. Conversely, among those uninterested in reducing their salt intake, such explicit labeling tended to be less preferred, whereas holistic wellness-oriented expressions such as “gentleness to the body” were preferred. In actual purchasing behavior, consumers cannot choose products independently of perceived product attributes such as taste, price, and safety; therefore, the extent to which packaging expressions influence purchasing behavior remains unclear. Furthermore, affective/experiential and cognitive/instrumental aspects are likely intertwined within individuals and may not be completely separable. Nevertheless, taken together, these findings suggest that individuals in the precontemplation stage who are indifferent to purchasing reduced-salt products may be particularly responsive to holistic wellness-oriented appeals that favorably stimulate the affective/experiential aspect of attitude toward such products.

Within the precontemplation stage examined thus far, there are individuals who are indifferent to purchasing reduced-salt products but still intend to purchase them. Previous studies have reported that the behavioral stage of salt reduction does not necessarily align with the willingness to purchase reduced-salt products, indicating that many people hold seemingly contradictory thoughts [36], consistent with our results. Furthermore, the research suggests that individuals can develop behavioral intentions and take action at lower stages without necessarily progressing sequentially through the behavioral stages [36]. Therefore, for those in the precontemplation stage, who constitute the majority of people not currently purchasing reduced-salt products, measures that foster positive affective impressions toward reduced-salt products may be promising targets for future interventions aimed at increasing purchase intention.

To answer RQ2, we calculated the percentage to gain using Hornik and Woolf’s method [24] and examined beliefs that may represent promising targets for future interventions. The results for the precontemplation stage are shown in Table 6. Top-ranked beliefs included: Belief item 9 “Using reduced-salt products gives me peace of mind that I am eating something that looks good for me,” Belief item 5 “I feel that by eating reduced-salt products, I can make my diet less taxing on my body,” and Belief item 44 “I want to consciously use reduced-salt products when I am feeling disordered in my diet.” These beliefs emphasize a holistic sense of wellness or general well-being rather than specific effects or symptoms. These findings suggest that holistic wellness-oriented beliefs emphasizing general health benefits may represent promising targets for individuals in the precontemplation stage. This is consistent with the aforementioned study’s finding that those uninterested in salt reduction prefer such expressions [13].

Furthermore, environmental factors were also shown to be important: Belief item 41 “I would buy reduced-salt products if they were available in the supermarkets I usually go to” or Belief item 48 “I would continue to use reduced-salt products if there were some benefits such as point rewards with purchase”. Belief item 41 suggests that availability in usual supermarkets may serve as an important environmental cue for purchase intention. In practice, supermarkets could apply this finding through choice architecture, for example, by placing reduced-salt products next to regular alternatives, improving shelf visibility, or using simple shelf tags that emphasize holistic wellness-oriented benefits rather than only explicit salt reduction. In addition, belief item 48 suggests that point-reward campaigns or similar incentives may provide another environmental support for encouraging consumers to try reduced-salt products.

Percentage gain is an estimate of the effect calculated from cross-sectional data at a single point in time and does not establish causality [24]; however, longitudinal studies have reported that beliefs identified as promising using this method predict subsequent behavioral changes [41]. Therefore, this is considered a useful method for selecting themes when considering intervention strategies.

When designing campaign implementations, planners must consider feasibility based on subjective judgments [24]. In this regard, the beliefs with the highest acquisition rates among men and women in the precontemplation stage were difficult to create through promotional campaigns. Therefore, when considering feasibility, designing experiences that foster a holistic sense of doing something beneficial for one’s health and establishing environments that enable such experiences may be a promising direction for future communication and food environment strategies.

This study’s limitations and directions for future research can be summarized as follows. First, this study’s participants were adults residing in Japan, and dietary culture, taste preferences, and primary sources of salt intake vary significantly by country and region. Therefore, caution is required when generalizing the results to other populations. Future research should validate these findings in populations with different dietary environments and cultural backgrounds.

Second, participants were recruited from online research panels, which may limit representativeness. Individuals registered with such panels may differ from the general Japanese population in that they may have greater access to the internet, higher digital literacy, and greater willingness to participate in surveys, as well as different socioeconomic characteristics. Therefore, the generalizability of the findings to all Japanese adults who are indifferent to reduced-salt products should be interpreted with caution.

Third, this was a cross-sectional survey conducted at a single time point and focused on purchase intention rather than actual purchasing behavior, salt intake, or health outcomes such as blood pressure. Therefore, further field or intervention studies are needed to examine whether strategies targeting the identified beliefs can increase actual purchases of reduced-salt products and contribute to reduced salt intake.

Fourth, the study focused on participants who were not actively purchasing reduced-salt products. Individuals in the contemplation stage or those in the action stage likely have different psychological attitudes toward reduced-salt product purchases compared to those in the precontemplation stage, who constituted the majority of the participants. Therefore, if the goal of social implementation is to encourage individuals currently uninterested in purchasing reduced-salt products to purchase them continuously, examining the different behavioral stages is also important.

Fifth, the relatively low internal consistency of the attitude construct should be acknowledged as a measurement-related limitation. Although an EFA was conducted to examine the internal structure of the attitude belief items, the attitude construct may include heterogeneous belief components. Therefore, findings related to attitude should be interpreted with caution. Future studies focusing specifically on the measurement structure of attitudes toward reduced-salt products should further examine their dimensionality using confirmatory factor analysis or related measurement models and refine the survey items accordingly.

Finally, this study examined attitude, perceived norm, and personal agency as independent psychological factors; however, human psychology is complex, and these factors may influence each other. Therefore, constructing a more precise verification model and further investigations are necessary in the future.

## 5. Conclusions

This study identified attitude as the IBM category most strongly associated with intention to purchase reduced-salt products among individuals who were not actively purchasing such products, particularly those in the precontemplation stage. The percentage-to-gain analysis further suggested that holistic wellness-oriented beliefs and product availability in usual supermarkets may be promising psychological and environmental targets for future communication and food environment interventions. By applying these targets to strategies such as holistic wellness-oriented communication and improved product availability in everyday shopping environments, these findings may help inform approaches that can be tested in future studies to promote actual purchases of reduced-salt products and reduce salt intake.

## Figures and Tables

**Table 1:** Summary of the belief items associated with the purchase of reduced-salt products, as clarified by the preliminary qualitative study.

Category		Item Count	Belief Items (No.)
Attitude	(positive)	9	1–9
	(negative)	11	10–20
Perceived norm		12	21–32
Personal agency		16	33–48

The terms “(positive)” and “(negative)” indicate the belief groups of the perceived positive and negative evaluation of reduced-salt products, respectively. The contents of belief items 1–48 are shown in Table S1.

**Table 2:** Characteristics and information regarding reduced-salt product purchasing behavior of primary quantitative questionnaire survey participants.

Characteristics		All (N = 800)		Men (N = 400)		Women (N = 400)		*p*-Value
		*n*	%	*n*	%	*n*	%	
Age (in years) [mean (SD)]		47.2	(8.6)	48.6	(8.1)	45.8	(9.0)	<0.001
Current behavioral stage for purchasing reduced-salt products								
	Precontemplation	649	81.1	335	83.8	314	78.5	0.071
	Contemplation	151	18.9	65	16.3	86	21.5	
Region of residence								
	Hokkaido and Tohoku	86	10.8	43	10.8	43	10.8	1.000
	Kanto	280	35.0	140	35.0	140	35.0	
	Chubu	134	16.8	67	16.8	67	16.8	
	Kinki	142	17.8	71	17.8	71	17.8	
	Chugoku and Shikoku	68	8.5	34	8.5	34	8.5	
	Kyushu and Okinawa	90	11.3	45	11.3	45	11.3	
Occupation								
	Company employee	314	39.3	215	53.8	99	24.8	<0.001
	Government employee	40	5.0	27	6.8	13	3.3	
	Self-employed/private business	65	8.1	50	12.5	15	3.8	
	Company officer	11	1.4	9	2.3	2	0.5	
	Medical professional	19	2.4	7	1.8	12	3.0	
	Homemaker	92	11.5	2	0.5	90	22.5	
	Student	10	1.3	3	0.8	7	1.8	
	Part-time job	152	19.0	25	6.3	127	31.8	
	Unemployed	90	11.3	58	14.5	32	8.0	
	Other	7	0.9	4	1.0	3	0.8	
Household size								
	1 person (the person lives alone)	192	24.0	117	29.3	75	18.8	<0.001
	2 people	217	27.1	90	22.5	127	31.8	
	3 or more people	391	48.9	193	48.3	198	49.5	
Household annual income								
	Less than 2 million yen	92	11.5	53	13.3	39	9.8	0.001
	2 million yen to less than 4 million yen	155	19.4	74	18.5	81	20.3	
	4 million yen to less than 6 million yen	110	13.8	55	13.8	55	13.8	
	6 million yen and more	251	31.4	144	36.0	107	26.8	
	Do not want to answer	192	24.0	74	18.5	118	29.5	
Chronic diseases other than hypertension								
	Present	73	9.1	39	9.8	34	8.5	0.624
	Absent	727	90.9	361	90.3	366	91.5	
Intention to purchase reduced-salt products								
	Present	267	33.4	116	29.0	151	37.8	0.011
	Absent	533	66.6	284	71.0	249	62.3	
IBM category score (points) [mean (SD)]								
	Attitude	3.06	(0.33)	3.04	(0.32)	3.09	(0.35)	0.049
	Perceived norm	2.98	(0.54)	2.93	(0.51)	3.03	(0.56)	0.013
	Personal agency	3.31	(0.68)	3.21	(0.65)	3.42	(0.68)	<0.001

SD, standard deviation; IBM, integrated behavioral model. Data are expressed as number of individuals (*n*) and percentage within each group (%), except for age and IBM category score which are expressed as mean and SD. The *p*-values represent the results of group comparisons conducted for each variable between men and women participants. The unpaired *t*-test was performed for comparisons of means. For comparisons of headcount distributions, the chi-square test or, in the case of 2 × 2, Fisher’s exact probability test was performed.

**Table 3:** Binary logistic regression analysis of the intention to purchase reduced-salt products.

IBM Category Score		OR	95% CI		*p*-Value
			Lower	Upper	
**Attitude**					
	Model 1	13.08	7.21	23.73	<0.001
	Model 2	12.56	6.93	22.79	<0.001
**Perceived norm**					
	Model 1	1.72	1.29	2.28	<0.001
	Model 2	1.67	1.25	2.23	<0.001
**Personal agency**					
	Model 1	3.74	2.74	4.87	<0.001
	Model 2	3.65	2.74	4.86	<0.001

IBM, integrated behavioral model; OR, odds ratio; CI, confidence interval. The dependent variable is intention to purchase reduced-salt products (1 = present, 0 = absent), with 0 = absent as the reference category. The values represent the results for each IBM category score, which is the primary explanatory variable. The analysis models were set as follows: Model 1: Each IBM category score individually; Model 2: Each IBM category score adjusted for age and gender. OR indicates how many times greater the odds of purchase intention are when the IBM category score increases by 1.

**Table 4:** Comparisons of the scores of the three IBM categories between the groups with and without intention to purchase reduced-salt products in the precontemplation and contemplation stages.

IBM Category Score	Behavioral Stage	Intention to Purchase Reduced-Salt Products				*p*-Value
		Absent		Present		
		Mean	SE	Mean	SE	
Attitude	Precontemplation	2.99	(0.01)	3.23	(0.03)	<0.001
	Contemplation	3.14	(0.07)	3.19	(0.03)	0.489
	All	2.99	(0.01)	3.21	(0.02)	<0.001
Perceived norm	Precontemplation	2.92	(0.02)	3.04	(0.05)	0.031
	Contemplation	3.21	(0.16)	3.13	(0.05)	0.632
	All	2.93	(0.02)	3.08	(0.03)	<0.001
Personal agency	Precontemplation	3.14	(0.03)	3.56	(0.05)	<0.001
	Contemplation	3.43	(0.16)	3.71	(0.05)	0.112
	All	3.15	(0.03)	3.64	(0.04)	<0.001

IBM, integrated behavioral model; SE, standard error. The number of individuals in the precontemplation/absence, precontemplation/presence, contemplation/absence, and contemplation/presence groups was 517, 132, 16, and 135, respectively. An unpaired *t*-test was performed for comparisons of means.

**Table 5:** Binary logistic regression analysis of the intention to purchase reduced-salt products in Precontemplation and Contemplation stage groups.

IBM Category Score		Precontemplation				Contemplation			
		OR	95% CI		*p*-Value	OR	95% CI		*p*-Value
			Lower	Upper			Lower	Upper	
Attitude									
	Model 1	18.60	8.60	40.26	<0.001	1.83	0.31	10.82	0.504
	Model 2	18.40	8.51	39.78	<0.001	1.60	0.24	10.53	0.622
Perceived norm									
	Model 1	1.54	1.05	2.24	0.025	0.79	0.33	1.90	0.600
	Model 2	1.50	1.03	2.19	0.036	0.70	0.28	1.73	0.437
Personal agency									
	Model 1	3.13	2.20	4.43	<0.001	1.91	0.89	4.09	0.097
	Model 2	3.08	2.16	4.38	<0.001	1.78	0.81	3.90	0.150

IBM, integrated behavioral model; OR, odds ratio; CI, confidence interval. The dependent variable is intention to purchase reduced-salt products (1 = present; 0 = absent), with 0 = absent as the reference category. The values represent the results for each IBM category score, which is the primary explanatory variable. The analysis models were set as follows: Model 1: Each IBM category score individually; Model 2: Each IBM category score adjusted for age and gender. OR indicates the number of times the odds of purchase intention increase when the IBM category score increases by 1.

**Table 6:** Top five percentage-to-gain outcomes for each belief item by gender in the precontemplation stage group.

Men			Women		
Belief Item	IBM Category	Percentage to Gain (%)	Belief Item	IBM Category	Percentage to Gain (%)
29	Perceived norm	20.2	6	Attitude	29.1
9	Attitude	17.6	44	Personal agency	19.4
41	Personal agency	16.9	9	Attitude	17.8
44	Personal agency	16.7	48	Personal agency	15.5
5	Attitude	15.9	41	Personal agency	14.6

The contents of each belief item are listed in Table S1.

## Data Availability

The data supporting the results of this study are available from the corresponding author upon reasonable request.

## Supplementary Materials

The following supporting information can be downloaded at: https://www.mdpi.com/article/10.3390/nu18111800/s1, Table S1: Belief items associated with the purchase of reduced-salt products, as clarified by the preliminary qualitative study; Table S2: Exploratory factor analysis of the 20 items comprising attitude; Table S3: Characteristics and the information regarding reduced-salt product purchasing behavior of the behavioral stage groups; Table S4: Characteristics of preliminary qualitative interview survey participants; Table S5: Sensitivity analyses excluding participants with chronic diseases other than hypertension; Table S6: Percentage to gain for all belief items by gender in the precontemplation and contemplation stages.
