# Supplementary material for: Psychological Determinants of Purchasing Behavior Among Individuals Indifferent to Reduced-Salt Products
Source: Nutrients. 2026 Jun 3;18(11):1800. doi: 10.3390/nu18111800 (PMC13259465; doi:10.3390/nu18111800)
Supplement: Supplementary file 1 [file nutrients-18-01800-s001.zip › nutrients-4316664-supplementary.pdf]

Supplementary Materials

Table S1. Belief items associated with the purchase of reduced-salt products, as clarified by the preliminary qualitative study

Category		Belief item	Content
Attitude	(positive)	1	I think that reducing salt intake will reduce the risk of high blood pressure.
		2	I think that reducing salt intake will reduce the burden on the kidneys.
		3	I think that reducing salt intake will reduce the likelihood of edema.
		4	I know from what I have seen and heard on TV and other media that too much salt can lead to high blood pressure.
		5	I feel that by eating reduced-salt products, I can make my diet less taxing on my body.
		6	When I was a child, I participated in events that educated people about salt reduction.
		7	I find it difficult to adjust the amount of salt in my daily diet on my own.
		8	I think food tastes good even if it is low in salt, as long as it has a good dashi flavor.
		9	Using reduced-salt products gives me peace of mind that I am eating something that looks good for me.
	(negative)	10	I would rather have good taste than good health.
		11	I have tried reduced-salt products in the past and felt they were not enough.
		12	I am concerned that reduced-salt products may contain more additives instead of less salt.
		13	In the case of reduced-salt seasonings such as miso and soy sauce, I feel that even reduced-salt products will end up having the same amount of salt as regular products if the amount of those products is increased.
		14	Since there is no numerical expression of how many grams of salt can be reduced in a reduced-salt product compared to a regular product, it is difficult to realize a reduction in salt.
		15	I feel that reduced-salt products are more expensive than regular products.
		16	I do not think that it is necessary to reduce salt in the first place, since the body needs it.
		17	I feel that there is not enough variety in reduced-salt products to choose from even if I wanted to.
		18	I feel that reduced-salt products are unnecessary except for those who are ill.
		19	I feel that reduced-salt products do not prevent disease.
		20	I don't want to change the taste of my home-cooking by using reduced-salt products.
Perceived norm		21	You have people close to you (*1) with whom you talk about each other's eating habits.<br>(*1) Below this question, "close" refers to people who are close to you, such as family members or close friends.
		22	Someone close to you is aware of having high blood pressure but is not interested in reducing salt.
		23	People close to you are more concerned about their intake of non-salt nutrients such as carbohydrates and proteins than they are about salt intake.

	24	Since everyone has different food preferences, I don’t think people should interfere with each other’s eating habits, even if they are close to each other.
	25	People close to you like strong flavors.
	26	Someone close to you prefers very strong flavors and you may be concerned about him or her.
	27	There are people close to you who can give you health advice based on their own experiences of illness.
	28	Someone close to you hates additives.
	29	There is someone close to you who is a salt-reduction oriented person.
	30	Many people close to you consider themselves not yet old enough to be conscious of salt reduction.
	31	I would rather preserve the taste of my own home-cooking style that I have had for a long time, rather than introduce reduced-salt products and arranging new home tastes on my own.
	32	I become more conscious of salt reduction when I am instructed to reduce salt during health checkups and other occasions.
Personal agency	33	If I try a reduced-salt product and there is no significant difference in taste from the regular product, I can continue to buy it.
	34	If the taste of reduced-salt products is acceptable to me, I can continue to use them.
	35	I can continue to buy reduced-salt products as long as there is not much difference in price compared to regular products.
	36	I can continue to buy reduced-salt products if they are less expensive than regular products.
	37	I may feel uncomfortable when I first switch to reduced-salt products, but I think I will get used to it and not mind it.
	38	If the additives used to make reduced-salt products are known to be safe, I can continue to use them.
	39	I just have not bought reduced-salt products before for no particular reason, and I would buy them if only there was some chance to do so.
	40	I would purchase reduced-salt products if I or my family were no longer healthy and needed them.
	41	I would buy reduced-salt products if they were available in the supermarkets I usually go to.
	42	If there are more types of reduced-salt products within a single product category (*2), I will be able to choose from among them and continue to buy them. (*2) Product category refers to a large group of products such as miso, soy sauce, instant noodles, frozen foods, and retort-pouch foods.
	43	I think I can continue to use reduced-salt products if I can gain health.
	44	I want to consciously use reduced-salt products when I am feeling disordered in my diet.
	45	I would consider purchasing reduced-salt products if I could see some benefit.
	46	I think I can continue to use reduced-salt products if they are used a few times a week instead of every day.
	47	I think I can continue to use reduced-salt products if other seasonings are used to add flavor.
	48	I would continue to use reduced-salt products if there were some benefits such as point rewards with purchase.

1–20: Attitude (1–9: Perceived positive evaluation of reduced-salt products; 10–20: Perceived negative evaluation of reduced-salt products); 21–32: Perceived norm; 33–48: Personal agency.

Table S2. Exploratory factor analysis of the 20 items comprising attitude

Belief item	Factor 1	Factor 2	Factor 3	Factor 4
2	**0.91**	0.00	-0.15	-0.14
1	**0.86**	0.02	-0.20	-0.07
3	**0.83**	-0.01	-0.18	-0.05
5	**0.79**	0.06	-0.14	0.20
4	**0.70**	0.06	-0.28	-0.05
9	**0.68**	0.05	-0.18	0.40
8	**0.56**	0.03	-0.31	0.07
18	0.13	**0.78**	0.08	-0.03
16	0.03	**0.75**	0.04	-0.07
19	0.13	**0.59**	0.12	-0.04
20	-0.10	**0.54**	0.21	-0.16
12	-0.15	**0.38**	0.32	-0.02
11	-0.10	**0.32**	0.25	-0.13
17	-0.23	**0.31**	0.30	-0.29
13	-0.29	0.10	**0.68**	0.23
14	-0.26	0.24	**0.66**	-0.05
15	-0.35	0.15	**0.58**	0.01
10	-0.16	0.28	**0.40**	-0.02
7	0.34	-0.02	**-0.37**	0.25
6	-0.08	-0.27	0.17	**0.55**

Extraction method: generalized least squares method. Rotation: Varimax rotation. All factor loadings are presented without suppressing small coefficients. The highest absolute factor loading for each item is shown in bold. Belief items 10–20 were reverse-coded before analysis, as described in Section 2.3.1. The content of each belief item is listed in Table S1.

Table S3. Characteristics and information regarding reduced-salt product purchasing behavior of the behavioral stage groups

Characteristics	Precontemplation (N=649)		Contemplation (N=151)		*p*-value
	n	%	*n*	%	
Age (in years) [mean (SD)]	47.4	(8.6)	46.7	(8.9)	0.391
Gender					
Men	335	51.6	65	43.0	0.071
Women	314	48.4	86	57.0	
Region of residence					
Hokkaido and Tohoku	70	10.8	16	10.6	0.900
Kanto	226	34.8	54	35.8	
Chubu	111	17.1	23	15.2	
Kinki	113	17.4	29	19.2	
Chugoku and Shikoku	53	8.2	15	9.9	
Kyushu and Okinawa	76	11.7	14	9.3	
Occupation					
Company employee	250	38.5	64	42.4	0.131
Government employee	35	5.4	5	3.3	
Self-employed/private business	52	8.0	13	8.6	
Company officer	9	1.4	2	1.3	

Medical professional	19	2.9	0	0.0	
Homemaker	67	10.3	25	16.6	
Student	7	1.1	3	2.0	
Part-time job	125	19.3	27	17.9	
Unemployed	79	12.2	11	7.3	
Other	6	0.9	1	0.7	
Household size					
1 person (the person lives alone)	156	24.0	36	23.8	0.681
2 people	180	27.7	37	24.5	
3 or more people	313	48.2	78	51.7	
Household annual income					
Less than 2 million yen	77	11.9	15	9.9	0.578
2 million yen to less than 4 million yen	127	19.6	28	18.5	
4 million yen to less than 6 million yen	84	12.9	26	17.2	
6 million yen and more	201	31.0	50	33.1	
Do not want to answer	160	24.7	32	21.2	
Chronic diseases other than hypertension					
Present	60	9.2	13	8.6	0.877
Absent	589	90.8	138	91.4	
Intention to purchase reduced-salt products					
Present	132	20.3	135	89.4	< 0.001
Absent	517	79.7	16	10.6	
IBM category score (points) [mean (SD)]					
Attitude	3.04	(0.33)	3.18	(0.30)	< 0.001
Perceived norm	2.94	(0.52)	3.14	(0.59)	< 0.001
Personal agency	3.23	(0.66)	3.68	(0.63)	< 0.001

SD, standard deviation; IBM, integrated behavioral model. Data are expressed as number of individuals (*n*) and percentage within each group (%), except for age and IBM category score which are expressed as mean and SD. The *p*-values represent the results of group comparisons conducted for each variable between precontemplation and contemplation stages. The unpaired t-test was performed for comparisons of means. For comparisons of headcount distributions, the chi-square test or, in the case of 2 × 2, Fisher's exact probability test was performed.

Table S4. Characteristics of preliminary qualitative interview survey participants

**Characteristics**	**All (N=48)**		**Precontemplation (N=24)**		**Contemplation (N=24)**	
	n	**%**	***n***	**%**	***n***	**%**
Age (in years) [mean (SD)]	41.0	(9.8)	41.1	(10.1)	40.8	(9.7)
Age category						
18–39 years old	24	50.0	12	50.0	12	50.0
40–59 years old	24	50.0	12	50.0	12	50.0
Gender						
Men	24	50.0	12	50.0	12	50.0
Women	24	50.0	12	50.0	12	50.0
Occupation						
Company employee	24	50.0	12	50.0	12	50.0
Government employee	3	6.3	1	4.2	2	8.3

Self-employed/private business	2	4.2	1	4.2	1	4.2
Company officer	1	2.1	0	0.0	1	4.2
Medical professional	0	0.0	0	0.0	0	0.0
Homemaker	7	14.6	4	16.7	3	12.5
Student	2	4.2	1	4.2	1	4.2
Part-time job	6	12.5	4	16.7	2	8.3
Unemployed	1	2.1	0	0.0	1	4.2
Other	2	4.2	1	4.2	1	4.2
Household size						
1 person (the person lives alone)	10	20.8	5	20.8	5	20.8
2 people	14	29.2	8	33.3	6	25.0
3 or more people	24	50.0	11	45.8	13	54.2
Household annual income						
Less than 2 million yen	2	4.2	1	4.2	1	4.2
2 million yen to less than 4 million yen	7	14.6	4	16.7	3	12.5
4 million yen to less than 6 million yen	6	12.5	3	12.5	3	12.5
6 million yen and more	30	62.5	14	58.3	16	66.7
I don’t know	3	6.3	2	8.3	1	4.2
Chronic diseases other than hypertension						
Present	9	18.8	5	20.8	4	16.7
Absent	39	81.3	19	79.2	20	83.3
Family members living in the same household with hypertension						
Present	24	50.0	12	50.0	12	50.0
Absent	24	50.0	12	50.0	12	50.0

SD, standard deviation. Data are expressed as number of individuals (*n*) and percentage within each group (%), except for age which is expressed as mean and SD.

Table S5. Sensitivity analyses excluding participants with chronic diseases other than hypertension

IBM category score	All				Precontemplation				Contemplation			
	OR	95% CI		*p*-value	OR	95% CI		*p*-value	OR	95% CI		*p*-value
		Lower	Upper			Lower	Upper			Lower	Upper	
Attitude												
Model 1	15.13	7.94	28.83	< 0.001	20.98	9.17	47.98	< 0.001	1.76	0.23	13.39	0.584
Model 2	14.71	7.72	28.05	< 0.001	20.99	9.18	48.03	< 0.001	1.46	0.17	12.21	0.728
Perceived norm												
Model 1	1.66	1.23	2.24	< 0.001	1.53	1.03	2.26	0.036	1.02	0.39	2.66	0.966
Model 2	1.63	1.20	2.20	0.002	1.47	0.99	2.19	0.056	0.95	0.35	2.61	0.927
Personal agency												
Model 1	3.72	2.77	5.00	< 0.001	3.16	2.19	4.55	< 0.001	2.18	0.98	4.84	0.056
Model 2	3.64	2.70	4.90	< 0.001	3.09	2.14	4.46	< 0.001	2.09	0.93	4.74	0.076

IBM, integrated behavioral model; OR, odds ratio; CI, confidence interval. The binary logistic regression analyses of the intention to purchase reduced-salt products were conducted after excluding participants who reported chronic diseases other than hypertension. The analysis groups comprised All (n = 727), those in the precontemplation stage (n = 589), and those in the contemplation stage (n = 138). The dependent variable is intention to purchase reduced-salt products (1=present; 0=absent), with 0=absent as the reference category. The values represent the results for each IBM category score, which is the primary explanatory variable. The analysis models were set as follows: Model 1: Each IBM category score individually; Model 2: Each IBM category score adjusted for age and gender. OR indicates the number of times the odds of purchase intention increase when the IBM category score increases by 1.

Table S6. Percentage to gain for all belief items by gender in the precontemplation and contemplation stages

Belief item	Percentage to gain (%)			
	Precontemplation		Contemplation	
	Men	Women	Men	Women
1	9.2	8.9	5.8	2.7
2	9.3	9.7	5.0	2.3
3	9.6	9.3	4.5	2.3
4	8.8	7.1	4.5	2.3
5	15.9	11.3	9.0	0.4
6	14.2	29.1	-19.5	8.1
7	9.4	7.4	-3.3	0.4
8	11.3	9.5	3.0	2.3
9	17.6	17.8	1.1	3.1
10 *	4.8	-1.5	-23.7	-10.6
11 *	5.6	0.8	8.0	1.5
12 *	2.4	3.0	3.8	-5.2
13 *	-9.1	-1.9	-6.2	-6.1
14 *	-2.4	-0.2	-11.2	8.1
15 *	-3.6	-6.3	-29.0	-6.1
16 *	2.9	9.2	4.3	-1.5
17 *	2.5	-2.9	-0.4	-0.2
18 *	15.9	13.3	-1.2	-1.4
19 *	6.4	13.0	-10.0	0.4
20 *	1.4	7.5	-7.6	-3.4
21	8.9	3.1	-2.8	2.1
22	1.8	3.8	3.3	1.7
23	7.8	8.3	-1.2	4.9
24	2.5	-4.8	1.3	-4.0
25	1.6	-0.6	6.2	1.0
26	7.9	5.6	2.1	0.4
27	7.1	6.9	-3.5	0.1
28	11.9	2.8	-1.2	-3.9
29	20.2	11.9	-11.2	1.2
30	1.4	6.6	0.5	-2.4
31	0.0	-0.3	-1.9	3.8

32	9.5	10.7	-0.9	3.0
33	6.7	8.9	0.9	1.5
34	7.5	6.6	3.0	1.7
35	8.2	9.0	4.2	2.0
36	7.4	7.6	6.7	1.4
37	8.9	9.9	4.5	-1.7
38	12.6	8.8	2.7	-0.8
39	15.4	12.9	2.1	-0.8
40	4.6	4.2	-1.3	2.1
41	16.9	14.6	5.0	1.5
42	15.4	14.0	0.9	1.4
43	13.0	11.7	6.2	-1.1
44	16.7	19.4	5.8	1.1
45	8.1	5.5	3.0	0.6
46	7.4	10.7	5.0	0.7
47	8.2	11.8	-2.4	-1.0
48	8.3	15.5	2.4	1.3

The content of each belief item is listed in Table S1.

* For the belief items 10–20, which correspond to perceived negative evaluation of reduced-salt products, the terms refer to the behavior change effect in the case where the entire target group were not to come to hold that belief. The number of individuals in the precontemplation/men, precontemplation/women, contemplation/men, and contemplation/women groups was 335, 314, 65, and 86, respectively.
